# User Expectations for Media Sharing Practices in Open Display Networks

**DOI:** 10.3390/s150716210

**Published:** 2015-07-06

**Authors:** Rui Jose, Jorge C. S. Cardoso, Jason Hong

**Affiliations:** 1 Centro Algoritmi, University of Minho, Guimarães 4800-058, Portugal; 2 CITAR/School of Arts, Portuguese Catholic University, Porto 4169-005, Portugal; E-Mail: jorgecardoso@ieee.org; 3 Carnegie Mellon University, 5000 Forbes Avenue, Pittsburgh 15213, PA, USA; E-Mail: jasonh@cs.cmu.edu

**Keywords:** locative media, location-based networks, social media, public displays, open display networks

## Abstract

Open Display Networks have the potential to allow many content creators to publish their media to an open-ended set of screen displays. However, this raises the issue of how to match that content to the right displays. In this study, we aim to understand how the perceived utility of particular media sharing scenarios is affected by three independent variables, more specifically: (a) the locativeness of the content being shared; (b) how personal that content is and (c) the scope in which it is being shared. To assess these effects, we composed a set of 24 media sharing scenarios embedded with different treatments of our three independent variables. We then asked 100 participants to express their perception of the relevance of those scenarios. The results suggest a clear preference for scenarios where content is both local and directly related to the person that is publishing it. This is in stark contrast to the types of content that are commonly found in public displays, and confirms the opportunity that open displays networks may represent a new media for self-expression. This novel understanding may inform the design of new publication paradigms that will enable people to share media across the display networks.

## 1. Introduction

Large screen displays are becoming an increasingly pervasive element of our technological landscape. Screens themselves are getting more affordable, but, at the same time, they are evolving to become more connected devices with the capability to integrate the digital eco-system around them. This technical evolution is creating entirely new expectations in regard to the role that screen displays may play as a communication medium. In particular, the emerging principles of Open Display Networks [1] suggest that future display networks should be open to content and applications from many sources. By decoupling content creation from display ownership, these large-scale networks of pervasive public displays would promote a many-to-many distribution paradigm in which content from many users would be shown wherever appropriate. This would represent a fundamental shift from today’s narrowcast models where content is centrally distributed and tightly controlled to an open model where user-generated content becomes a major source of display media.

While previous research has already studied many variants of user-generated content for public displays [2,3,4], such research has mainly assumed a publication scenario in which content is posted to a specific display within a clear context of “here and now”. In these cases, the context in which that media will be shown is implicitly defined by that particular situation and its strong locativeness [4], *i.e.*, the assumption that media representing the service relates to its immediate physical environment. Appropriateness is directly linked to the interpretation made by the publisher about the current display setting, and any social negotiation surrounding the shared used of the display is normally implicit in the interaction process itself. However, when we consider media sharing across an open-ended set of displays, this association with a specific context is lost. The challenge is then how to redefine appropriateness, beyond the context of “here and now”, *i.e.*, how to match the content being posted by people anywhere on the network to the displays that may provide a meaningful context for its presentation.

Like many other forms of communication, including social networking services, media sharing on a public display occurs within the scope of a wider social context that frames the notion of what might be appropriate to present. We thus envision that meaningful communication over open display networks may be considerably enhanced if these networks were able to reflect some of the social connections in which communication is naturally inscribed. A proper choice of connections should be able to preserve the context of the content and protect a display in a highly interconnected and global network of displays from unrelated content. A social graph promoting social interactions around content published on public displays should thus address three fundamental goals: allow the publisher to express relevance, allow the display owner to assess that relevance and support collaboration.

In this study, we aim to understand user expectations regarding media sharing across large networks of public displays. More specifically, we explore how the perceptions of appropriateness can be affected by the nature of the content and the scope of publication. We consider the effects of three independent variables in the perceived utility of media sharing scenarios, more specifically: (a) the locativeness of the content being shared; (b) how personal that content is and (c) the scope in which it is being shared. To assess these effects, we composed a set of 24 media sharing scenarios embedded with different treatments of our three independent variables. These scenarios were inspired by common media sharing scenarios from various social networking platforms, so that they could seem familiar to participants and represent meaningful media sharing situations. We then asked 100 participants to express how likely it would be that they would share media in the situations depicted by each of the scenarios.

The results suggest a strong preference for those situations in which the displays are used for posting content that is both local and directly related to the person that is publishing it. This is in stark contrast to the types of content that are commonly found in public displays, and confirms the opportunity that open displays networks may represent a new media for self-expression. The main contribution of this work is this novel understanding of how different properties of the media sharing scenarios may impact the perceived value of those scenarios. This may inform the design of new publication paradigms for open display networks that enable people to share media across the whole network.

## 2. Related Work

This work relates with prior work on user-generated content on public displays, and also with work on media sharing practices on location-based social networks.

### 2.1. User-Generated Content on Public Displays

A very broad range of techniques has been studied to enable display systems to accept content originating from users. One of the earlier examples, the Plasma Poster [2], allowed people to submit photos, text, and web pages to a public display using email or a web form. The Short Message Service (SMS) and the Multimedia Messaging Service (MMS) have also been extensively used as an interaction technique for the spontaneous generation of content. For example, the Joe Blogg project [5] includes a display designed in the form of an interactive artwork where people can send pictures and text messages through MMS or SMS. Hermes [6] explored the use of Bluetooth to enable users to send pictures and other media to a display. The use of Bluetooth names have been described in [4,7], as an essentially opportunistic alternative for enabling interaction and user-generated content on a broad range of mobile devices. The possibility to abstract multiple forms of input has been explored in [8] and would enable common interaction practices to emerge, regardless of the specific technologies involved. These, and many similar research studies, have mainly addressed particular interaction procedures that enable content to be pushed by people to the displays. In this work, we aim to address the publication of user-generated content from a broader perspective, which is more focused on enabling people to understand and control what happens after content reaches the displays.

Despite the many techniques for placing user-generated content on public displays, Huang and Mynatt [9] observed that individuals tend not to be motivated to supply content, or else have difficulty identifying appropriate content. Similarly, Müller *et al.* [10] describe how public displays may be perceived as a stage on which people will only act if they feel confident about their actions and in full control over the presentation of self. To a certain extent, any form of user-generated content for public displays is always embedded with some type of publication paradigm that drives the conceptual model behind the process. In previous work, these publication paradigms have been implicit within the properties offered by particular applications, such as mediating socialization in public spaces [11,12], sharing content of interest to a group of people [12], supporting collective music selections around the display [13] or selecting themes for image presentations [3]. This approach is strongly coupled with the semantics of specific applications and does not provide a generalizable paradigm that users can learn to use in many different displays and settings. Memarovic [14] has studied privacy concerns associated with situated snapshots that are then shown on multiple locations. While we do not specifically address the issue of privacy, this study is relevant for our work because it demonstrates how people may raise many types of concerns when faced with a media distribution process over which they have no control, even if they control the content generation process.

More general publication practices around large scale networks of public displays have been studied by Storz *et al.* [15] in a long-term analysis of the e-campus deployment at Lancaster University. This work only considered the view of display managers and confirmed the diversity of requirements that different stakeholders, even in a single organization, may have in regard to display control and the way content should be published. These findings highlight the importance of flexible publication tools that people may easily appropriate to support very diverse content publication practices. Publication practices are also a central topic for Instant Places [16], an open network for public displays that allows people to systematically manage content publication. The system has been deployed across a set of locations, at which participants were allowed to create and distribute digital posters for presentation on public displays. While the system does not explicitly support friends or any other sort of social connections, participants in the study have expressed their willingness to create such connections, e.g., media sharing partnerships between places and membership type of connection between places and selected people. This suggests that social connections can play a key role in the perception of content appropriateness and in supporting new forms of socially-mediated content exchanges. Melro *et al.* studied media sharing and moderation practices associated with the distribution of leaflets in coffee houses [17]. The results indicate that media acceptance criteria are very different between venues and far more sophisticated than what may be anticipated by simplistic rules of appropriateness.

Altogether, these findings suggest that at least part of the challenges involved in making user-generated content a reality are not directly related with the interaction process itself. Instead, they seem to be more strongly associated with the motivation, the context and the meaning of the media sharing process. They also show that people resort to very diverse types of formal and informal connections to express what they see as being the proper publication scope for a particular screen media item. Our aim is to uncover in more detail what these types of connections might be and how different types of content can be perceived differently.

### 2.2. Bridging between Physical and Online Social Networks

Social networks are now prevalent on the Internet and increasingly they are also embracing the activities of people in the physical world. The wide availability of mobile data and smart devices makes it very easy or even automatic to associate location information with all sorts of user actions and share that information with existing online social networks.

Location-based Social Networks include location information into their social graph to enable users to see where their friends are, to search location-tagged content within their social graph, and to meet others nearby [18]. This ability to gather and manipulate real world contextual data that bridges the gap between the physical world and online social networking services represents new opportunities for technology mediated social interactions and an interesting context for intelligence gathering.

As location is one of the most important components of user context, extensive knowledge about an individual’s interests, behaviors, and relationships with others can be learned from visited locations. For some of these networks, such as Foursquare or Google Latitude, location is not just there to enhance already existing interactions. Location is the real core business of those services and the social object around which everything else happens.

The relation between physical co-presence and on-line social friendships has been studied by Cranshaw *et al.* [19], who showed that such relations are strongly dependent on the entropy of the locations visited and the number of social ties that a user has in the network. The ways in which different types of interpersonal relationships may be associated with the willingness to share information between people has been studied by Wiese *et al.* [20]. The results show that the willingness of people to share content with others on their social networks is associated with the perception of closeness in relation to that person but also with the frequency of communication and to a lesser extent in scenarios with common information, e.g., being physically proximate. Scellato *et al.* [21] have also studied the effect of frequency of the same places on link prediction, *i.e.*, the likelihood that two people will become friends in a social network. Using data from a location-based social network, the results show that 30% of the new links are added among “place-friends”, *i.e.*, among users who visit the same places.

Despite all the research interest around social networks, relatively little has been done in examining the role of social networks in the context of user-generated content for public displays. Part of our conjecture here is that a user’s choice to publish a given piece of content would be influenced by understanding who might see that content, and the relationship between the user and the places where that content could potentially be shown. While sharing some of the same properties of location-based social networks, particularly the key role of location and the use of presence as expressed through the physical proximity to the displays, a social network for open display networks would have to support a new type of social graph that is anchored on places and their meaning as communication contexts.

The notion of what might be appropriate to present at any particular display requires a shared understanding about social relevance that cannot be defined a priori. From this perspective, the problem is in many ways similar to social networks, and the ways in which they explore multiple forms of social graphs to convey meaning and define the appropriateness of media sharing. A display network that was embedded with social connections should thus be able to provide context to user-generated content, driving it to the displays where its presentation would be more relevant, for both the publisher and the viewers. Our work is an initial step towards understanding these media sharing situations and the types of connections that can be used to add social meaning to the process.

## 3. Methodology

In this research, we seek to uncover the key elements that people perceive as more relevant for expressing and interpreting appropriateness when sharing media across an open network of public displays. We assume that the central social object is a screen media item that someone, called the publisher, intends to post for presentation across an open network of public display. It is around this social object that social engagement should emerge, such as re-distributing, rating, commenting or collecting screen media items. Content on a screen media item may have been created by the person herself or simply pulled out from some third-party external source and packaged for presentation on displays. In either case, we assume that screen media items cannot be changed after being posted to the display network, so that a reputation can be established based on their content.

### 3.1. Independent Variables

Our research methodology is anchored on the perceived utility associated with different scenarios of media sharing across large networks of public displays. Our research design is focused on understanding how that perception of utility is affected by three independent variables, more specifically: (a) the locativeness of the content being shared; (b) how personal that content is and (c) the scope in which it is being shared.

In regard to locativeness, we consider the extent to which content is related to a local scope. We aim to understand the relevance that locative media may play in future display networks. In particular, the balance between locative and global content is determined by the opposite nature of these types of media. Highly locative media may be favoured at the places where it is relevant, but because it is only relevant in a limited context, it will never reach as many displays in the network as a globally relevant content.

We also consider the effect of how personal the content is. Considering the public nature of the displays, our notion of personal does not include any privacy–sensitive content. Instead, we are just considering authorship or the extent to which the content is an expression of identity. Like in most social networks, a media item may have been created by the person herself to express personal views or it may have been created by re-sharing an already existing media item that was pulled from an external source.

Finally, we also consider how different sets of places can provide meaningful contexts for expressing the scope of media sharing. We assume that when posting screen media items to the display network, publishers will be asked to express their view of the respective publication scope, *i.e.*, the set of places where the presentation of that content is seen as appropriate by the publisher. Whether or not the item will actually be presented at particular displays may depend on the policies of those displays, but this expression of scope represents the publishers’ view of where the content should be considered for presentation. Unlike what is common in social networks, expressing the scope of media sharing is not about privacy. Since content may potentially be seen by anyone passing by any of the displays where it is being shown, limiting the range of displays where it will be shown cannot be seen as a way to prevent content from being seen by unintended audiences. Our view of the role of the publication scope is therefore much more focused on identifying where it might be more relevant to show that content, rather than restricting its presentation to the places where it can be shown.

### 3.2. Scenario Specification

A major challenge for this research is that open display networks are not yet a reality that is part of people’s everyday lives. This means, in the first place, that we do not yet have any infrastructures from which to obtain data about existing media sharing practices. It also means that it is harder to ask people to anticipate their behaviour in regard to situations that they have never experienced before. To mitigate these challenges, we devised a study anchored on a set of carefully designed scenarios inspired by common media sharing situations from social media that we re-purposed for the context of open display networks. These scenarios have been designed around two major requirements: they should seem as natural as possible to people, even to someone who may have never have heard of display networks before; and they should represent the key research questions of the study, by being embedded with properties that correspond to our key independent variables.

To comply with the first requirement, we started by defining six media sharing situations inspired by common practices from social media. The goal was to minimize bias on possible content types and also to have scenarios that provided, as much as possible, a familiar frame of reference for participants. We selected a set of popular services with diverse properties in regard to their goals and media sharing practices, more specifically Facebook, Twitter, Pinterest, Craiglist and Causes. For each of these services, we searched for content rankings and identified the types of content that were shared the most. We then pruned the results to exclude content that would clearly not make sense on public displays, either for privacy reasons or because of the nature of the content itself. The result was a list with over 30 specific examples of media sharing in social media.

To comply with the second requirement, we selected a sub-set of six sharing situations that were representative of two of the independent variables in our study, more specifically, the variables regarding the local and the personal nature of the content being shared. We started by analyzing the media sharing examples and classifying them according to those two independent variables of our study. We then selected six situations that seemed more appropriate for our specific scenario and represented different combinations of our independent variables, as shown on Table 1.

**Table 1:** The six base stories embedded with different locality and personal properties.

ID	Description	Locality	Personal
**1**	A funny video of a dance	Global	No
**2**	A Garage sale announcement	Local	Yes
**3**	Photos of new IPhone launch	Global	No
**4**	Food at local restaurant	Local	Yes
**5**	Poster World AIDS Day	Misc	No
**6**	Missing dog appeal	Local	No

These six stories include situations in which the content being shared is potentially relevant on a global scale, e.g., a funny dance video, and other scenarios where the relevance of the content is much more local, e.g., the missing dog. The world AIDS day was not considered for this variable because it was ambiguous in the sense that it was a global campaign with local initiatives that could easily be interpreted both ways. Similarly, the stories also include situations in which content being shared is not at all personal, e.g., the iPhone launch, and situations where content involves a personal form of expression, e.g., sharing a good experience at a local restaurant.

Each situation was then described in the form of a short story adapted to fit the specific circumstances of public displays. The goal was to allow people to identify themselves with the essence of the media sharing context and motivations. These stories described the whole context of the media sharing situation, clearly stating, not just the type of content but also the intentions associated with sharing:

(1) *A funny video of a dance*: “Someone shares on Facebook a video of a wedding in which the bride and her father make a very nice dance together. You think this is a very touching moment and at the same time a very funny video that will look great on a public digital display and put a smile on the face of whoever may happen to see it.”
(2) * The garage sale announcement*: “You are redecorating your house and as part of that process you now have considerable furniture and old stuff that you want to sell. You decide to organise a garage sale and create a digital poster announcing the event.”
(3) *The new iPhone launch*: “You receive a tweet about how the launch of a new iPhone model is drawing crowds to stores around the world. The tweet includes a link to nice pictures depicting those crowds. You think this is timely and interesting information.”
(4) *Food experience at a local restaurant*: “You have been eating out on a nice new restaurant and you really enjoyed their fabulous goat cheese salad. When it was brought to you, it looked so nice that you took a photo. At the end, you decide to publish that photo together with your comment about the nice experience you have just had.”
(5) *World AIDS Day*: “It is World AIDS Day and all over the world, there are initiatives exploring what AIDS means to different communities. A high school in your city is promoting a fund raising initiative and you decide to make your personal contribution by reusing content from their media announcements to publish a simple digital poster appealing to the cause.”
(6) *The missing dog*: “Your neighbour has just lost his dog and he is very concerned about what might happen. You decide to help by creating a digital poster with the photo of the dog and a contact number that people may call if they find it.”

To complete the scenario specification, we still needed to introduce the other independent variable of our study: the media sharing scope. This defines the strategy that people can use to express where it will be more meaningful to show the content that they are sharing on the display network.

We assumed this scope to correspond to a set of places expressed through an intentional definition. We thus ruled out, at least for the purpose of this study, the possibility to indicate specific individual displays. Instead, we focused only the case where the display set is expressed in the form of implicit or explicit connections that people could have with places.

For identifying meaningful sets of places, we consider a socio-spatial graph composed of two types of nodes (people and places) and their respective connections. People are the participants that will engage in content publication. Places represent the locations where the displays are deployed and where content may potentially be shown. We chose places as nodes, rather than displays, because there might be many displays on a single place and also for considering that the concept of place translates better into the social and administrative dimension that is needed to support meaningful social connections. We then considered multiple variants of the socio-spatial graph connecting people with places. A key property in this social graph is the strongly locative nature of the displays, and thus the major role that physical presence may play in shaping the graph. Many place groups may emerge naturally from previous physical proximity and locative interactions with displays. These are implicit connections that result from interactions that are not meant to create any connection. For example, people currently at the same location, people that have visited a location before, or people that often go to the same places are all natural associations that implicitly result from the independent behaviour of multiple people. Based on these associations, many types of connections with display groups can be implicitly defined and used for establishing media sharing relevance. Still, contextual relevance is also very much about social meaningfulness, not just physical proximity. Therefore, other forms of connection should also be supported to capture these sources of social relevance, e.g., places explicitly marked as favourite.

For this study, we selected four types of connections between people and potential places for media sharing. Table 2 represents these connections, which together represent various types of social ties, both implicit and explicit, and different strategies for content distribution.

**Table 2:** Media distribution strategies to be combined with base stories.

ID	Scope Definition	Mode
**A**	Share in the places most visited by friends.	Implicit
**B**	Share in the places marked as favourite.	Explicit
**C**	Share in previously visited places.	Implicit
**D**	Share where you are a frequent visitor	Implicit

Finally, we combined these four media distribution strategies with each of the six base stories to obtain the final set of 24 media sharing that we used for our study. These 24 stories are embedded with different treatments of our three independent variables.

### 3.3. Evaluation of the Perceived Utility of the 20 Stories

Our experimental setting was thus composed by 24 stories representing scenarios of media sharing in display networks. Each story was a unique combination of our three independent variables, more specifically, the locativeness of the content (global or local), the personal nature of content (personal or not), and the distribution strategy used for expressing the media sharing scope (A, B, C, D).

The dependent variable is the perceived value that participants associate with each of the media sharing scenarios. To gather this data, we run a survey on Amazon’s Mechanical Turk, with workers located at the USA. We divided the 24 scenarios into four different evaluation tasks, each consisting of a survey where participants were asked to evaluate how likely was it that they would publish content to a network of public displays in the same way as described in each of the media sharing scenarios. Their answers ranked from 1 (not at all likely) to 5 (very likely). Each task was composed by a subset of six of the 24 scenarios, but we selected them in a way that all the six base stories (see Table 1) were present on each task. To reduce bias, the order of the scenarios on each task was randomized.

We ran four evaluation panels consecutively, over a period of a month. They were all launched at about the same time of the day and their average duration was 6.5 days. The panel size (30) was larger than the number of respondents we eventually selected (25), so that we had some margin to discard recurrences. The larger panel size was also useful for discarding evaluators that did not execute their task in a responsible manner. For identifying these cases, we have followed each scenario evaluation with a verification question to ensure that respondents were paying the appropriate attention to their task. A total of 112 participants answered the four evaluation panels. From these, we discarded eight survey responses done by recurrent evaluators or in which there was evidence of lack of a responsible job. At the end, we randomly discarded four others to get the same number of results per scenario and ended up with 100 survey responses by unique participants expressing 600 opinions about the proposed scenarios, and more specifically 25 evaluations on each of our 24 scenarios.

## 4. Results and Discussion

The results of this study are grounded on the 100 validated responses obtained from participants. A higher result means that participants perceived the scenario as corresponding to something that they were more likely to do. Overall, the Missing Dog and the Garage Sale scenarios were the three scenarios that were consistently rated as being the most likely. However, our analysis is mainly focused on assessing the effects of our study variables on the sub-set of scenarios that correspond to the different treatments of our experience, as represented in Table 3.

**Table 3:** Means and standard deviations of the responses.

Variable	Level	Mean	SD
**Locality**	Local	3.56	1.32
	Global	2.42	1.30
**Personal**	Personal	3.40	1.32
	Non-personal	2.98	1.42
**Distribution strategy**	Dist. A	2.96	1.42
	Dist. B	3.23	1.34
	Dist. C	3.11	1.44
	Dist. D	3.17	1.41

The table shows the mean and standard deviations of the participants’ responses for each of those treatments and respective levels. The characteristics that differentiate the treatments are the locality of content (two levels), the personal nature of the content (two levels) and the distribution strategy (four levels).

**Figure 1:**
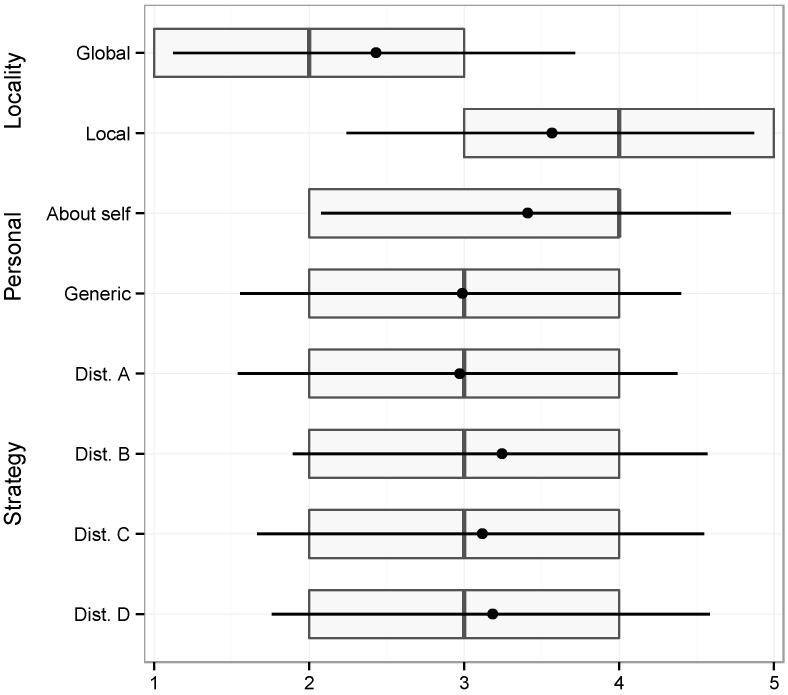
Boxplots of the responses for the various variables and levels.

These same results are also depicted in Figure 1, in the form of boxplots for the various variables and levels. The left and right sides of the boxes represent the first and third quartiles, respectively, and the line inside the box represents the median. Given the nature of our data, with discrete values and all the scenarios having the same minimum (1) and the same maximum (5), we decided to overlay information about the mean value (the dot) and one standard deviation below and above the mean.

These results suggest that participants make a very strong association between the locative nature of content and its relevance for publication across public displays. We will now analyze in more detail the results for each of the three elements that composed the independent variables in our study.

### 4.1. Locativeness

One of the goals was to identify the effect of locativeness on the perceived value of the media sharing scenarios. From the six base stories, there were two (funny video of a dance in a wedding and Photos from the new IPhone launch) that represented content with potentially global scope and three that represented content with local scope (food suggestion at local restaurant, missing dog and garage sale). We excluded the World AIDS Day scenario from this analysis because of the ambiguity between local events by the local community and those events being part of a world day.

The main result is that participants have clearly favoured content that was local in scope. The scenarios in which content is more locally relevant stand out very clearly in the boxplot as being the ones for which there is a more positive perception of relevance. To verify the statistical significance of these results, we ran a one-way ANOVA test between the two groups (local and global). The results confirm the existence of a statistically significant effect of locality on the perceived value associated with the sharing situation (F1, 498 = 90.54, *p* < 2e-16).

These results and also the strength of the observed effect suggest that participants make a very strong association between the locative nature of content and its relevance for publication across public displays.

### 4.2. Personal Content

Our second goal was to identify the effect of the personal nature of content being published to the display network, on the perception of relevance. From the six base scenarios, there were two scenarios (garage sale announcement and food at local restaurant) that were directly about the publisher. The other four scenarios (funny video of a dance; photos from new IPhone launch; poster on World AIDS Day and missing dog) represented content that referred to others.

This variable follows a behavior similar to locality. Even though the box plots for personal content seem less distinct, the median and mean values for personal content are clearly more positive than those for not-personal content. A one-way ANOVA test on the two groups of scenarios (personal and non-personal) confirms that the personal nature of content also has a statistically significant effect on the perceived relevance associated with the sharing situation (F1, 598 = 12.3, *p* = 0.000486).

Participants see more relevance in sharing content that refers to them instead of content that is simply about others. None of the scenarios, however, was really personal information in the sense of information that normally would only be disclosed to a known set of friends. The garage sale is typically an announcement that needs to be advertised to the community and the food suggestion, although implying the expression of a personal preference, is also a way of informing the community about something that may genuinely be of interest to others. We thus conclude that participants seem to find more value in the possibility to display content that directly relates to their reality.

### 4.3. Distribution Strategy

A key issue when considering publication of content across a display network, rather than to a particular display, is the extent to which people are still able to express the scope of the publication act.

Regardless of the reach of a publication act, the fact that people actually express the scope of that publication could be very relevant to assess the relevance of a particular content item for a particular place. We wanted to observe to what extent people would be sensitive to the four distribution strategies embedded in the media sharing situations.

The box plots for the distribution strategy show no obvious difference among the various strategies. A one-way ANOVA test on the four groups of scenarios corresponding to the four types of distribution strategies (F3, 596 = 1.052, *p* = 0.369) indicates that we cannot confirm any statistically significant effect of the distribution strategy on the perceived relevance.

Possibly, participants did not have a strong idea about these forms of content distribution or they may simply have failed to make any meaningful distinction between them. All of our distribution strategies were, to some extent, local, as they all implied regular physical presence to the places where the displays were located. Therefore, in any of the scenarios, the scope of publication, even if composed by very different sub-sets of displays, was inherently local and seen as appropriate.

Still, the only distribution strategy that was based on an explicitly formed group of displays (those marked as favourite) was the best-ranked one. Even though our results cannot confirm the statistical significance of these findings, they seem to suggest a tendency towards a more explicit control over the set of displays where media is shared.

## 5. Conclusions

In this study, we analysed the perceived value of different scenarios of media sharing in open display networks. We have considered the effects of two types of content properties, more specifically, how local and how personal the content is, and also the effect of the social connection of the publisher with the places where content may be shown. The main result is a clear preference for content that is both local and personal. When interpreted through a social media mind-set, this may seem, at first, as an obvious result. However, this is in stark contrast to the types of content that today can be commonly found in most public displays. This seems to confirm the idea that future open displays networks, where everyone can have some possibility to publish content, are likely to revolve mainly around situated content that is fundamentally different from what we have today in current digital signage systems [1].

We have not identified statistically significant effects with regard to specific distribution strategies. Still, participants have preferred the only alternative in which the publication scope is expressed as an explicitly defined set of places. This seems to suggest that, even though content is not private and people are sharing it to gain visibility, there is still a strong sense of appropriateness in relation to potential presentation places. Future display networks should thus consider the issue of how to enable people to effectively keep control over the scope of publication.

These results may inform the design of new publication paradigms for public displays that go beyond the context of immediate publication to a single display and are able to explore multiple, open-ended and relevant publication opportunities that may arise across an entire network of public displays. In our future work, we intend to explore new types of connections between publishers and displays to assess alternative models to create a meaningful relationship between screen media items and display opportunities.
